# Urea Amidolyase (*DUR1,2*) Contributes to Virulence and Kidney Pathogenesis of *Candida albicans*

**DOI:** 10.1371/journal.pone.0048475

**Published:** 2012-10-29

**Authors:** Dhammika H. M. L. P. Navarathna, Michail S. Lionakis, Martin J. Lizak, Jeeva Munasinghe, Kenneth W. Nickerson, David D. Roberts

**Affiliations:** 1 Laboratory of Pathology, Center for Cancer Research, National Cancer Institute, National Institutes of Health, Bethesda, Maryland, United States of America; 2 Fungal Pathogenesis Unit, Laboratory of Clinical Infectious Diseases, National Institute of Allergy and Infectious Diseases, National Institutes of Health, Bethesda, Maryland, United States of America; 3 National Institute of Neurological Disorders and Stroke, National Institutes of Health, Bethesda, Maryland, United States of America; 4 School of Biological Sciences, University of Nebraska, Lincoln, Nebraska, United States of America; New Jersey Medical School, University of Medicine and Dentistry of New Jersey, United States of America

## Abstract

The intracellular enzyme urea amidolyase (Dur1,2p) enables *C. albicans* to utilize urea as a sole nitrogen source. Because deletion of the *DUR1,2* gene reduces survival of *C. albicans* co-cultured with a murine macrophage cell line, we investigated the role of Dur1,2p in pathogenesis using a mouse model of disseminated candidiasis. A *dur1,2*Δ*/dur1,2*Δ strain was significantly less virulent than the wild-type strain, showing significantly higher survival rate, better renal function, and decreased and less sustained fungal colonization in kidney and brain. Complementation of the mutant restored virulence. *DUR1,2* deletion resulted in a milder host inflammatory reaction. Immunohistochemistry, flow cytometry, and magnetic resonance imaging showed decreased phagocytic infiltration into infected kidneys. Systemic cytokine levels of wild-type mice infected with the *dur1,2* mutant showed a more balanced systemic pro-inflammatory cytokine response. Host gene expression and protein analysis in infected kidneys revealed parallel changes in the local immune response. Significant differences were observed in the kidney IL-1 inflammatory pathway, IL-15 signaling, MAP kinase signaling, and the alternative complement pathway. We conclude that Dur1,2p is important for kidney colonization during disseminated candidiasis and contributes to an unbalanced host inflammatory response and subsequent renal failure. Therefore, this *Candida*-specific enzyme may represent a useful drug target to protect the host from kidney damage associated with disseminated candidiasis.

## Introduction

*Candida albicans* is the most prevalent fungal pathogen of humans. It can be isolated from ca. 30% of patients in intensive care units [1], and patients with disseminated candidiasis have mortality rates of 30–40% [2]. *C. albicans* is a formidable opportunistic pathogen in part due to its metabolic and morphological flexibility, ability to adapt to different locations within the body, and rapid shifting from commensal colonization of the gastrointestinal tract to being an invasive pathogen [3], [4]. Investigation of fungal virulence factors has identified important roles for genes that regulate yeast to hypha switching, phenotypic switching from white to opaque cells associated with mating, biofilm formation, adhesion towards epithelial cells, and a variety of extracellular enzymes.

Our laboratories have focused on identifying novel virulence factors including farnesol [5], heme oxygenase [6], and urea metabolism via urea amidolyase [7]. Urea is an end product of human nitrogen metabolism, and thus provides a nitrogen source that *C. albicans* can exploit without competing with its host for nutrients. Urea catabolism is known to contribute to virulence of bacterial pathogens such as *Helicobacter pylori* [8] and *Proteus mirabilis* [9]. Urea degradation is also exploited by some fungal pathogens. The metalloenzyme urease that hydrolyses urea is a well known virulence factor for *Cryptococcus neoformans*. [10] and enhances pathogenesis of Coccidioidal infections [11]. Deletion of urease significantly reduced brain infection and pathology of mice and significantly increased host survival [12]. Therefore, it is pertinent to investigate urea catabolism and virulence of *C. albicans*.

The higher fungi exhibit a dichotomy with regard to urea utilization. Like all hemiascomycetes (yeasts), *C. albicans* uses the energy requiring bifunctional enzyme urea amidolyase (Dur1,2p), whereas other higher fungi use the nickel-containing urease [13]. The enzyme urea amidolyase, encoded by *DUR1,2* (Degradation of URea), was first characterized in the yeast *Candida utilis* [14]. This cytoplasmic, biotin dependent enzyme consists of a single protein chain with domains for both urea carboxylase and allophanate hydrolase activity. We made a *dur1,2*Δ*/dur1,2*Δ mutant (KWN6) and the complemented strain *dur1,2*Δ*:: DUR1,2/dur1,2::DUR1,2* (KWN8) [15]. The mutant strain KWN6 was unable to use urea as a nitrogen source and unable to escape from macrophages [15]. Dur1,2p is a cytoplasmic enzyme and gains access to urea primarily via the transporter Dur3, encoded by *orf 19.781*, which is co-regulated with Dur1,2p [7]. Examination of the evolutionary origins of urea amidolyase by comparing 64 fungal genomes and 56 bacterial genomes revealed that the urea amidolyase genes currently found in fungi likely are the result of a horizontal gene transfer event ca. 400 mya from the beta- or gamma- proteobacteria [16].

The present paper compares the *C. albicans dur1,2*Δ*/dur1,2*Δ mutant (KWN6) and reconstituted strains with their wild type parent A72 with regard to virulence in mice, fungal burden, inflammatory cell infiltration into infected kidneys, and triggering of local and systemic host inflammatory gene expression. Infection with KWN6 without urea amidolyase led to moderate kidney colonization, less inflammation in the kidneys, improved kidney function, and better survival of the host. Identification of urea amidolyase as a virulence factor for *C. albicans* further implies that the distribution of urea within the body influences the organ tropism of *C. albicans*. In addition, Dur1,2p expression alters local inflammatory responses leading to clearance of the respective organs and local and systemic cytokine responses that regulate host immunity against *C. albicans*.

## Materials and Methods

### Ethics Statement

Experimental protocols, housing, and care of mice were conducted in an AAALAC approved facility according to animal study protocol LP-022 approved by the National Cancer Institute Animal Care and Use Committee.

### Strains and growth conditions

The *C. albicans* strains used for this study are A72 WT strain (ATCC MYA-2430) KWN6 (*dur1,2*Δ*/dur1,2*Δ), KWN7 (*dur1,2*Δ*/dur,12*Δ*::DUR1,2*), and KWN8 (*dur1,2*Δ*::DUR1,2/dur,12*Δ*::DUR1,2*) [15]. For challenge of mice, *C. albicans* cells were grown overnight in 50 mL of Yeast Peptone Dextrose (YPD) medium at 30°C with aeration as previously described [17]. Cells were harvested by centrifugation at 5000 rpm for 10 min, washed once with 50 mL of sterile, non-pyrogenic normal saline (Quality Biological Inc. Gaithersburg, MD) and resuspended in 10 mL of saline before quantifying cell numbers using a Petroff-Hausser counting chamber. The cell suspensions were adjusted to the final concentration for parenteral administration using non-pyrogenic sterile saline.

### Mouse inoculation with *C. albicans*

Six to eight week-old (18–20 g) BALB/c female mice (Charles River Laboratories, Wilmington, MA) were randomly allocated to groups of five animals. Each group of mice was inoculated intravenously in the lateral caudal tail vein using a 27 gauge needle with a volume of 0.1 ml containing 10^6^ *C. albicans* cells [5], [17]. Clinical signs of illness in each mouse were evaluated three times daily, and mice that displayed severe signs were euthanized immediately by placing them in a closed chamber filled with CO_2_ and processed for complete necropsy and collection of tissues for histopathological examination. To longitudinally monitor effects of *C. albicans DUR1,2* on serum cytokines and chemokines, organ burden and host immune responses, mice were euthanized sequentially from 6 h to 240 h post-inoculation (PI). A total of 40 mice were inoculated with A72, 40 were inoculated with KWN6, and 10 control mice received no fungal challenge. Three animals from each group were sacrificed at 6, 12, and 24 h and then every other day until seven day PI for histopathology and cytokine assays. The 10 control animals, i.e., untreated and uninfected, were sacrificed, and the organs and sera were collected. The mean results for these 10 control animals were used as time zero values. Sera separated from the blood collected from individual mice were stored at −80°C until analysis.

To reproduce the CFU study, histopathology, IHC, and flow cytometry, five mice per group were infected with *C. albicans* and euthanized at specific time periods for sample collection. Mice of 3 days PI were used For MRI studies to image phagocytic cell infiltration and conduct histopathology and IHC. Ultra-small particles of iron oxide (USPIO) were injected 24 h before MRI. Clinical signs of illness were monitored four times daily whilst subjected to MRI procedures.

To analyze renal function and validate NanoString gene expression data using qRT-PCR, two groups of mice were infected with WT (A72) and KWN6 (*dur1,2*Δ*/dur1,2*Δ*). Non infected mice were used as negative controls. At 3 days PI the mice were euthanized, and blood was collected for serum kidney function markers analysis and organs for RNA extraction.*

For analysis of local inflammatory gene expression, two groups of mice consisting of 5 mice per group were infected with A72 wild-type and KWN6. Mice were euthanized at 3 day PI, and kidneys were harvested for RNA extraction.

### ^14^C-Urea uptake

*C. albicans* cells were grown overnight in 50 ml of YPD, washed and resuspended in PBS, and used as the inocula (0.2 OD) for fresh cultures then grown in GPP (glucose phosphate proline medium) at 30°C for 3–4 h while shaking at 150 rpm. Cell numbers were counted and adjusted so that all uptake assays employed an equal number of cells. Assays were done in 5 ml of GP (glucose phosphate) buffer in 14 ml BD tubes (BD Biosciences, NJ) with or without 5 mM sodium azide, using ca. 0.5×10^8^ cells and 1 μCi per 1.15 μg of ^14^C-urea (American Radiolabeled Chemicals, St. Louis, MO) per ml (19.2 µM urea). Cells incubating with ^14^C-urea were shaken at 150 rpm, and duplicate 0.2 ml samples were collected at 5, 15, 30, 60, and 120 min. A 0.2 ml sample was overlaid on 100 μl of 30% sucrose in 0.4 ml microfuge tubes without caps and centrifuged for 30 s, repeated with the second 0.2 ml sample, and the top 0.3 ml was aspirated off to remove unbound radioactive urea, whereupon 300 μl of PBS was added, and the cells were centrifuged again before aspirating all liquids leaving the yeast pellet. The bottom piece of the microfuge tube was cut off, added to scintillation fluid, mixed by shaking, and radioactivity was quantified by scintillation counting.

### Organ burden quantification

Three mice from each group were euthanized at day 1, 3, 5 PI to determine the fungal burden in their kidneys. At the time of necropsy, kidneys were harvested from each mouse and placed in sterile Eppendorf tubes. The tissues were kept at 4°C until the next day, when each kidney was weighed and homogenized in 1.0 ml of nonpyrogenic sterile saline. Then, 10 fold serial dilutions of 10^−2^, 10^−4^ and 10^−6^ in 0.1 ml of the homogenates were spread on triplicate plates of Nickerson's medium, also known as BiGGY agar, a selective and differential medium for *C. albicans* [18]. After 48 h of incubation at 30°C, colony number, morphology, and color were recorded, and numbers of CFU per kidney were estimated. *C. albicans* appears as brown to black colonies with no pigment diffusion and no sheen [18].

### Necropsy and Histopathology

Immediately after euthanasia, macroscopic changes were recorded, and the brain, heart, lungs, liver, spleen, and right kidneys were immersed in buffered 10% formalin, processed for paraffin embedding, sectioned at 5 μm, and stained with haematoxylin and eosin (H&E). Grocott's modification of Gomori's methenamine-silver (GMS) stain was used for detection of fungi in situ [19]. Histopathology images from sections of formalin-fixed and paraffin-embedded tissues stained with Gomori's methenamine-silver or H&E were obtained using a light microscope (Olympus BX51) fitted with a digital camera (Nikon DXM1200F) and ScanScope XT digital scanner (Aperio). Images were processed with Adobe Photoshop and Aperio ImageScope v11.1.2.760 (Aperio).

### Immunohistochemistry

Slides were deparaffinized in xylene (thrice for 10 min) and rehydrated in graded alcohol (100%, 95%, and 70%). Antigen retrieval was performed in a jar containing Target Retrieval Solution (pH 6.10; Dako Corp.) for 20 min, followed by cooling at room temperature for 20 min, and then washed with PBS twice for 10 min. Endogenous peroxidase activity was quenched by incubating with peroxidase block (pretreatment of the tissue section with hydrogen peroxide prior to incubation of primary antibody) for 30 min to avoid Endogenous peroxidase activity. After washing the slides with Wash Buffer Solution (Dako), nonspecific binding was reduced using Protein Block Serum-Free (Dako) for 10 min. The slides were incubated with anti iNOS antibody (1∶50, overnight at 4°C, ab15323). Slides were then incubated with streptavidin-biotin (Dako LSBA+ kit, horseradish peroxidase). 3,3′-Diaminobenzidine (Dako) was used as chromogen for 5 min, and hematoxylin was used for counterstaining. Negative control slides omitted the primary antibody. iNOS was located predominantly within the cells. Nuclei were negative. The intensity of the staining was evaluated using light microscopy and Adobe Photoshop.

### MRI procedures

Mice were anesthetized in an induction chamber with a 30% oxygen/70% nitrogen (oxygen-enhanced air) gas mixture containing 5% isoflurane. After anesthesia was induced (indicated by loss of righting reflex, decreased respiratory rate and non-responsive to toe pinch), isoflurane was reduced to 1.5–2%, and the animals were maintained via a nose cone.

For MR imaging of the region of interest, the body of the anesthetized mouse was restrained in a plastic holder with either vet wrap or tape. Care was taken to allow for chest expansion to facilitate breathing. A respiratory sensor pillow was placed under the mouse or attached above to monitor respiratory rate and pattern. MRI may be performed for up to 3 hours; however a typical anatomical scan was approximately 1 hour or less.

Macrophage infiltration of the kidneys was observed using ultra small particles of iron oxide (USPIO). Using a gradient echo sequence (TR/TE  = 200/10 ms, FOV  = 50×32 mm, matrix  = 256×256, 4 averages) T2*-weighted images of the kidneys were acquired 3 days post inoculation, 24 hours following i.v administration of a USPIO contrast agent Molday ION_TM_ (Bio PAL, MA) 0.1 μl suspended in 10 μl of saline(0.2 μmol of iron per kg body weight).

### Kidney inflammatory cell quantification by flow cytometry

To quantitatively define the inflammatory cell infiltration in the infected kidneys, the animals were perfused with normal saline and organs were harvested. Single-cell suspensions were obtained, and flow cytometry analysis was performed as reported previously [20]. Briefly, to obtain kidney single-cell suspensions, the organs were finely minced and digested at 37°C in digestion solution containing 0.4 mg/ml of Liberace CITL and 300 U/ml of grade II DNAse I for 20 min with intermittent shaking. Digested tissue was passed through a 70 μm filter, washed, and the remaining red cells were lysed with ACK lysing buffer for 30 s. Then, the cells were passed through a 40 μm filter, washed, and suspended in 8 ml of 40% Percoll. The suspension was overlaid on 3 ml of 70% Percoll and centrifuged at 2,000 rpm for 30 min at RT. The leukocytes at the interphase were isolated, washed three times in FACS buffer, suspended in PBS, and passed through a 40 μm filter. Suspensions of 100 μl were fixed with 100 μl of 2% paraformaldehyde (USB, Cleveland, Ohio, USA) for leukocyte quantification using PE-conjugated fluorescent particles (Spherotech, Lake Forest, Ill., USA). The cells were stained with a Live/Dead fluorescent dye (Invitrogen, Carlsbad, Calif., California, USA) for 10 min (1∶500) and then incubated with rat anti-mouse CD16/32 (2.4G2; BD Biosciences, San Jose, California, USA) for 10 min (1∶100) at 4°C to block Fc receptors. Then, cells were incubated at 4°C for 30 min with the following antibodies: FITC-conjugated anti-mouse Ly6c (AL-21, BD Biosciences) and CD19 (1D3; eBioscience, San Diego, California, USA); eFluor® 450-conjugated anti-mouse MHC II (I-A/I-E; M5/114.15.2) and CD3 (17A2); eBioscience,); PE-conjugated anti-mouse Ly6G (1A8) and NK1.1 (PK136; BD Biosciences), and MHC II (M5/114.15.2; eBioscience, San Diego, Calif., USA); PE-Cy7-conjugated anti-mouse CD45 (Ly-5; BD Biosciences), CD8 (53-6.7), and F4/80 (BM8; eBioscience); APC-conjugated anti-mouse CD45 (Ly-5),) and CD11c (HL3; BD Biosciences), and CD19 (1D3; eBioscience); APC-Cy7-conjugated anti-mouse CD11b (M1/70) and CD3 (17A2; CD45 (Ly-5; BD Biosciences), and APC-fluor-780-conjugated anti-mouse CD4(RM4–5; eBioscience). After three washes with FACS buffer, cells were fixed with 2% paraformaldehyde. FACS was performed on an LSRII (BD Biosciences), and data were analyzed using FlowJo software (version 8.8.4; Treestar, Ashland, Oreg., USA). Quantification of the leukocyte subsets in the infected kidneys was performed using PE-conjugated fluorescent particles (Spherotech, Lake Forest, Ill., USA) as previously described [20].

### Determination of serum cytokines and kidney protein markers

Murine serum was collected from sacrificed mice at various time points following infection with wild- type and mutant *C. albicans* strains. A Luminex -bead array (Mouse cytokine/Chemokine LINCOplex Kit, catalog no. 551287, Linco Research, Inc. St Charles MO) was used for detection of the cytokines IL-1a, IL-4, IL-6, IL-10, IL12, IL17, and IFN-γ, and TNF- α, and G-CSF according to the manufacturer's specifications. IL-7, IL-15, MIP-1α, MIP1β, and MIP-2 levels of kidney extracts and serum were analysed using the Luminex bead array Milliplex MAP Kit (catalog no MPXMCYTO-70K, Millipore, Billerica, MA).

### NanoString gene expression analysis

Expression of specific mRNAs in total kidney RNA was analyzed using the NanoString methodology as previously reported [21], and was conducted at the DNA sequencing core facility of NIH. Briefly, 100 ng of total RNA per kidney were hybridized to the target specific mouse inflammatory gene Codeset at 65°C.

The Codeset contained probes against a panel of 179 genes encoding proteins involved in mouse inflammation and six internal reference genes and were used to analyze local inflammatory response in kidneys. The hybridized reactions were loaded onto the NanoString Prep station, which removes excess reporter, binds the reporter to the cartridge surface, and stretches the probes for scanning. Subsequently, the cartridges were loaded onto the NanoString Digital Analyzer and scanned.

An Excel-based method described by the manufacturer was used to perform normalization to six internal controls and basic statistical analysis on the data. The normalized results are expressed as the relative mRNA level, and values for kidneys infected with WT and DUR1,2 deleted strain were averaged and are presented as mean ± s.d. Statistical significance was calculated using Student's t-test and was set as p<0.05. Genes that were statistically significant and showed >2.5-fold higher or lower expression are listed in the Table 1. Using MetaCore™ pathway analysis software from Genego [21] up- and down-regulated genes clusters were analyzed for association with specific signaling pathways and direct interactions.

**Table 1:** Inflammatory gene expression in infected mouse kidneys by NanoString analysis.

Gene	p value	F C	gene	p value	F C	Gene	p value	F C
Nos2*	0.0153	54.2	Il10rb	0.0002	−2.6	Rps6ka5	0.0249	−5.1
Ccl3*	0.0000	42.1	Keap1	0.0024	−2.6	Kng1	0.0034	−5.2
Il1rn	0.0205	37.1	Hmgn1	0.0004	−2.7	Prkca	0.0019	−6.1
Fos*	0.0454	26.6	Nfe2l2	0.0003	−2.8	Il7	0.0046	−6.5
Maff	0.0000	24.2	Mknk1	0.0004	−2.8	Traf2*	0.0225	−7.2
Ccl4	0.0050	18.2	Mapkapk5	0.0031	−3.0	Mef2a	0.0292	−7.6
Il18rap	0.0002	17.9	Map3k7	0.0023	−3.0	Plcb1	0.0155	−7.6
Il1a	0.0120	15.8	Tlr3	0.0152	−3.0	Fxyd2	0.0437	−7.7
Tnf	0.0004	14.5	Map2k4	0.0022	−3.2	Nfatc3	0.0054	−8.0
Il1b	0.0096	14.1	Nr3c1	0.0196	−3.4	Ly96	0.0005	−8.6
Itgb2	0.0446	12.6	Raf1	0.0100	−3.5	Map2k6*	0.0160	−9.7
Cebpb	0.0151	9.1	Gnaq	0.0009	−3.6	Il15*	0.0260	−13.5
Il8rb	0.0413	9.0	Map3k5	0.0048	−4.1	C8a*	0.0234	−193.2
Hspb1	0.0341	8.9	C2	0.0007	−4.2			
Tlr2	0.0008	6.5	Creb1	0.0217	−4.3			
C3	0.0097	5.8	Lta	0.0035	−4.3			
Limk1	0.0178	3.9	Mef2c	0.0122	−4.3			
Cxcr4	0.0095	2.8	Tgfb2	0.0328	−4.7			
Tgfb1	0.0055	2.7	Stat1	0.0011	−4.9			

F C – fold change in mRNA expression in kidneys of mice 3 days after infection with A72 wild type versus the *dur1,2*Δ strain KWN6. * indicates selected genes validated with qRT-PCR (Figure S1).

### RNA Extraction and Gene expression analysis by RT-PCR

RNA isolation was done using a standard hot phenol procedure [22]. Reverse transcription was conducted using 5 µg of total RNA extracted from each sample using Superscript III reverse transcriptase according to the manufacturer's instructions for oligo-dT priming (Invitrogen). Quantitative PCR was conducted as previously described [6] using Absolute QPCR SYBR Green Mix (Thermo Scientific), Opticon I instrument, and Opticon I software (BioRad). Samples were analyzed by PCR in triplicate and normalized to internal CDC36 mRNA levels. Melting curve analysis was performed to assure a single product was produced in each reaction [6]. The qPCR primers used in this study are designed using Primer3 software [23] and listed in Table S1, and all products were 100–150 bases in length.

### Statistics

The probability of survival as a function of time was determined by the Kaplan-Meier method, and significance was determined by the log-rank (Mantel-Cox) test and Jehan-Breslow-Wilcoxon test using GraphPad Prism software. The mixed procedure of the SAS system [24] was used to analyze serum cytokine expression patterns among all treatment groups at various time points. Three randomly selected mice from each group were euthanized at each time point for longitudinal comparisons. Data were analyzed for significant differences by comparing means of each triplicate reading at various time points assuming that the cytokine expression levels within each group of mice are normally distributed [25]. Fungal burden and ^14^C urea absorbance were analyzed using Student's t test. qRT-PCR gene expression data were analyzed by one-way ANOVAs using GraphPad Prism software.

## Results

### Validation of the urea amidolyase null mutant

**Figure 1:**
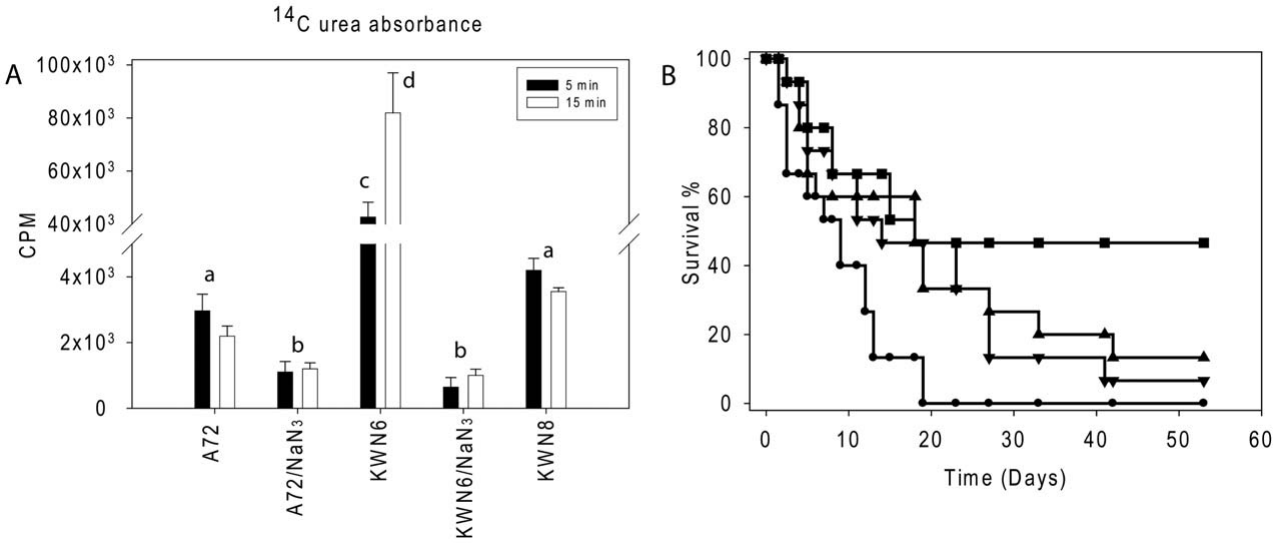
Dur1,2p function in urea metabolism and virulence. (A)**.** Urea uptake by WT (A72), *dur1,2*Δ*/dur1,2*Δ mutant and reconstituted *C. albicans* strain *dur1,2*Δ*::DUR1,2/dur1,2*Δ*::DUR1,2.* Radioactive uptake into 4−5×10^6^ cells is presented after exposure to ^14^C urea for 5 and 15 min in the absence or presence of sodium azide. Values shown are the average of triplicate experiments ± SE. Closed bars indicate 5 min uptake, and open bars indicate 15 min uptake of radioactive urea. (B)**.** Effect of *DUR1,2* deletion on mouse mortality following intravenous infection. Survival of mice injected with WT *C. albicans* A72 (•), the null mutant KWN6 (▪), single copy reconstituted KWN7 (▾), fully reconstituted KWN8 (▴), and an uninfected control group of 5 mice (not shown) was assessed daily. Each infected group contained 15 mice.

The null strain KWN6 was constructed from A72, a wild type *Candida albicans*, as part of our study of arginine and its metabolite urea in the escape of *C. albicans* from the mouse macrophage cell line RAW264.7 [15]. KWN6 could not use urea as the sole source of nitrogen, could not make germ tubes in response to either arginine or urea, and could not escape from the mouse macrophage cells. All of these abilities were restored in the reconstituted strain KWN8. In contrast, KWN6 grew as well as its parent on all other complex and defined media tested [15]. Collectively, these observations indicated that Dur1,2p is necessary for urea metabolism in *C. albicans* [15]. Further confirmation that urea amidolyase activity had been eliminated from KWN6 was provided by assessing ^14^C-urea uptake by the respective strains (Fig. 1A). The absence of Dur1,2p in KWN6 reduces but does not eliminate induction of the major urea transporter Dur3p in the presence of urea [7]. Despite its relative deficit in urea transport activity, cellular accumulation of urea via Dur3p or other putative urea transporters was ca. 20- to 40-fold greater for KWN6 than for the A72 parent (Fig. 1A). Thus, intracellular urea accumulation is primarily limited by its catabolism by Dur1,2p. Urea uptake in the reconstituted KWN8 returned to levels close to those of the wild type parent, confirming that urea metabolism is restored in this strain (Fig. 1A). As observed for *S. cerevisiae* [26], urea transport is an energy dependent process, and the elevated intracellular urea accumulation in KWN6 was prevented in the presence of sodium azide (Fig. 1A). These experiments confirm the physiological function of urea amidolyase *in vitro* and show that energy dependent urea transport remains active in the urea amidolyase null mutant.

### *DUR1,2* deletion reduces virulence in mice

The well-established mouse model of disseminated candidiasis was used to compare virulence among the WT and manipulated strains. Mice infected with the *dur1,2*Δ*/dur1,2*Δ strain (KWN6) had a significantly higher survival rate compared with mice infected with the WT A72 strain (n = 15, p<0.01, Fig. 1B). The Gehan-Breslow-Wilcoxon test hazard ratio estimates indicated 3.4-times higher lethality for WT infection than KWN6. Restoration of one copy (KWN7) or both copies of *DUR1,2* (KWN8) restored virulence but not fully to the level of the WT parent (Fig. 1B, P<0.01). However, by 7–8 weeks the survival of mice infected with the A72, KWN7 and KWN8 strains did not differ significantly.

### Disruption of *DUR1, 2* impairs kidney and brain colonization

**Figure 2:**
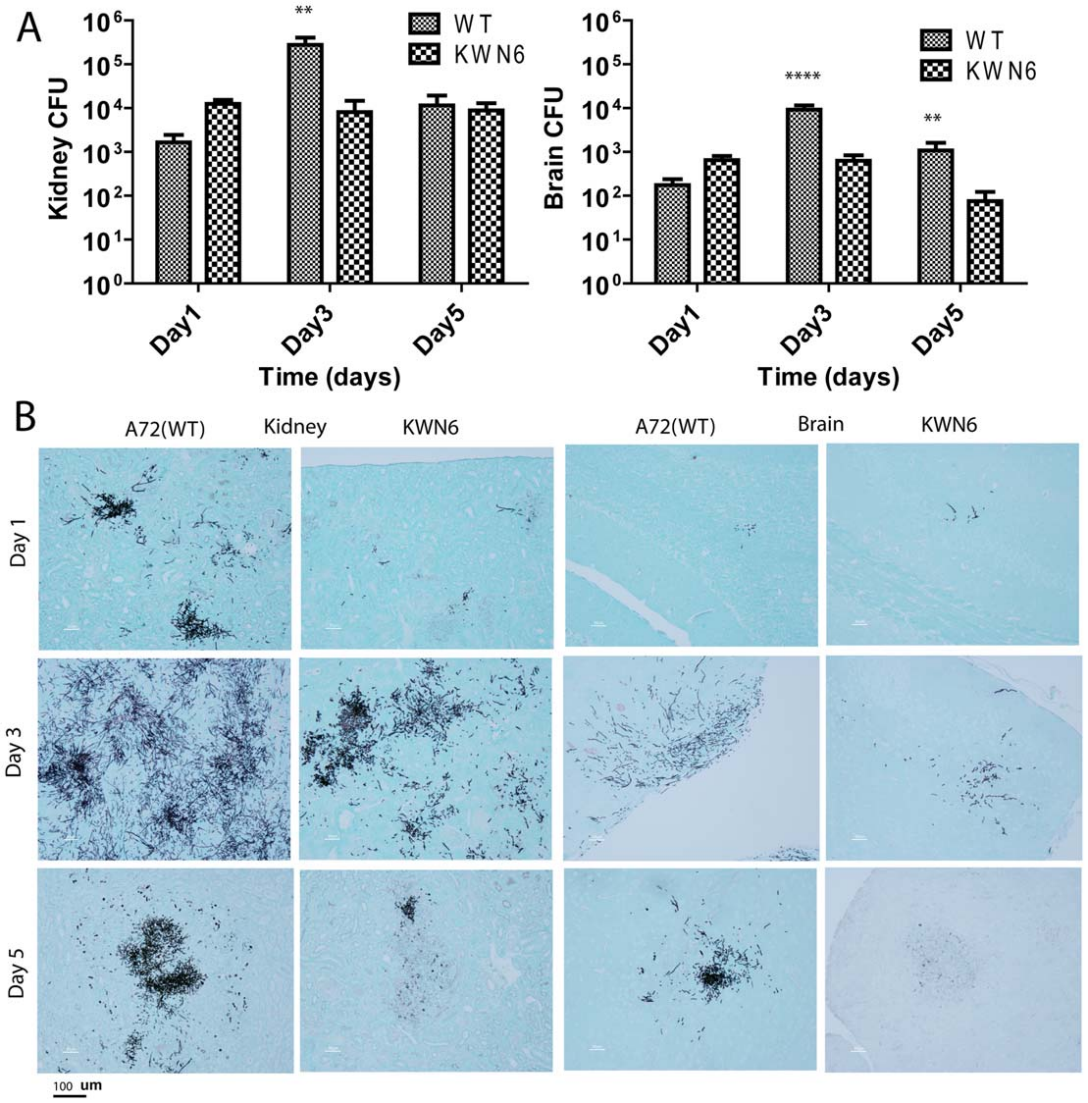
Dur1,2p expression promotes kidney and brain colonization. (**A**)**.** Kidney and brain fungal burdens of mice infected with parental A72 and mutant KWN6 strains. Gray bars represent mean CFU of WT infected left kidney homogenates determined by CFU on BiGGY agar from six representative serial dilutions from each kidney, representing three mice per time point infected. Checkered bars represent similar mean values for mice administered with KWN6. (B) GMS-stained mouse kidney and brain tissues to determine *C. albicans* colonization. Representative kidney and brain sections harvested day 1, 3 and 5 PI from mice infected with WT or KWN6 are shown. Scale bar indicates 100 μm.

Urea is present in serum at 70–150 mg/L [27], at high concentrations in the kidneys and brain [28], [29], and much lower concentrations in other organs [30]. If *C. albicans* uses urea as a nitrogen source *in vivo*, the virulence defect observed in Fig 1B could arise from Dur1,2p-dependent colonization of organs containing high levels of urea. Consistent with this hypothesis, the fungal burdens for A72 and KWN6 were dramatically different in the mouse kidneys (Fig. 2A left) and brain (Fig. 2A right). Analysis of fungal CFU at 3 days PI showed 15-fold (p<0.01) and 12-fold (p<0.001) lower CFU in kidneys and brains, respectively, of mice injected with the urea amidolyase null mutant (Fig. 2A).

CFU values can underestimate organ burden for filamentous fungi, so we used GMS staining to confirm the role of urea catabolism in determining the fungal burden (Fig. 2B). Colonization was reduced at all time points in mice infected with the urea amidolyase null mutant KWN6. Differences were observed in both kidneys and brain (Fig. 2B). At 1 day PI, *C. albicans* colonies were scattered throughout the cortex of kidneys infected with wild type *C. albicans,* while the KWN6 infected group showed minimal colonization (Fig. 2B). By day 3 PI, WT infected kidneys showed extensive fungal invasion (Fig. 2B left), as did the brain tissue (Fig. 2B right). In both cases the WT *C. albicans* had a higher fungal level compared with the mutant infection. Hyphal filaments were more abundant in kidneys infected with WT, but the mutant did show limited filamentous growth in the kidney.

Longitudinal GMS staining was used to visualize the clearance of fungi from the organs by the mouse immune system. *Candida* colonization was reduced or eliminated in the brain and the kidney cortex of KWN6 infected mice after 5 days, whereas the WT infected mice failed to clear these colonizations (Fig. 2B). As reported previously [20], [31], *Candida* colonization during pathogenesis gradually shifted from the cortex to the kidney pelvis starting around day 3 PI, and by day 7–9 PI the WT *C*. *albicans* formed large fungal balls in the pelvis, whereas the KWN6 strain showed only mild colonization (data not shown). Beyond day 9 PI, we did not observe *Candida* colonies in KWN6 infected mice kidneys, whereas surviving WT infected mice maintained *Candida* colonies at 17 days PI (data not shown). After initial colonization, *C. albicans* is quickly cleared from liver, spleen and heart by the mouse immune system, whereas kidney and brain are sites of chronic colonization [20], [49]. Our results suggest that colonization in kidney and brain cannot be sustained in the absence of Dur1,2p.

### Disruption of *DUR1,2* preserves kidney function

**Figure 3:**
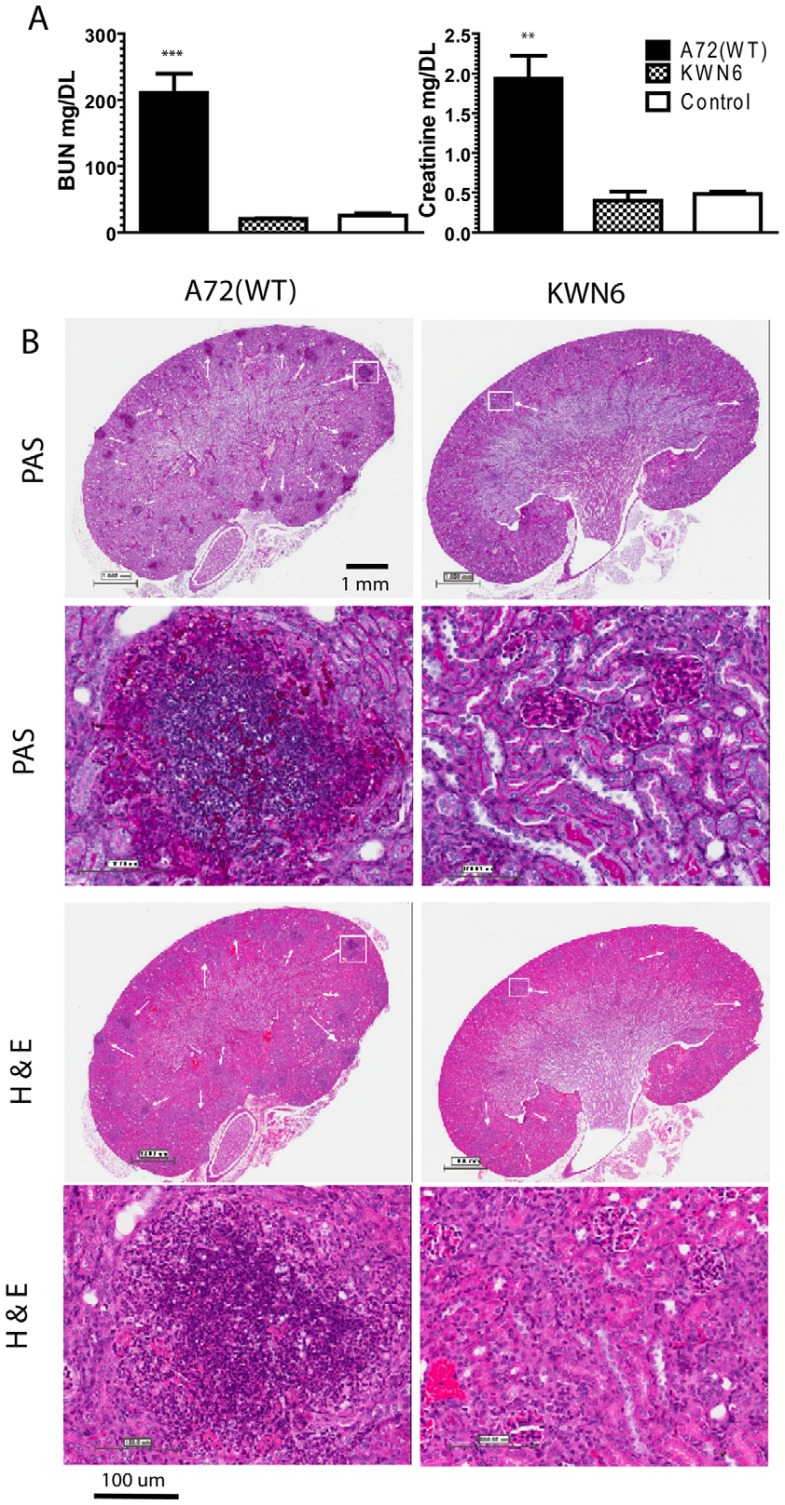
Dur1,2p expression impairs kidney function and increases inflammation. **A**. Kidney function tests of mice infected with A72 (WT) vs KWN6 at day 3 PI. Open bars represent serum levels of BUN and creatinine in non-infected control mice. Checkered bars represent KWN6 infected mice, and closed bars represent A72 (WT) infected mice. Note the levels of BUN and creatinine are significantly higher in WT infected sera compared with KWN6 infected mice. B. PAS and H&E-stained mouse kidney tissues to assess inflammatory responses and tissue necrosis caused by *C. albicans* colonization. Representative sections of infected mouse kidneys (group of 3 mice) harvested at day 3 PI are shown. Scale bars indicate magnification. Arrows indicate regions of PMN and *C. albicans* accumulation in PAS stains and PMN inflammatory foci in H&E stains. Demarcated regions by white squares are shown under higher magnifications in the lower panels.

Mice infected with WT *C. albicans* had significantly impaired kidney function compared with either uninfected mice or KWN6 infected mice as determined by their elevated blood urea nitrogen (BUN) and creatinine levels (Fig. 3A). The kidney damage indicated by these markers was 10- and 5-fold greater, respectively, in the WT parental strain than the urea amidolyase null mutant (Fig 3A). WT infected mice at 3 day PI had BUN  = 210±50 mg/dL vs KWN6 infected mice which had 20±1.2 mg/dL (p<0.002), while the serum creatinine levels of WT infected mice were 1.9±0.5 mg/dL vs KWN6 infected mice, which had 0.4±0.2 mg/dL (p<0.01).

### Dur1,2p regulates the kidney inflammatory response

Consistent with the greater fungal colonization of the kidneys at day 3 PI (Fig. 2), PAS and H&E stained kidney sections at day 3 PI showed that disruption of *DUR1,2* decreased the inflammatory response in infected kidneys at 3 days PI for mice infected with KWN6 (Fig. 3B). No signs of inflammation were observed 6–12 h PI, but by day 1 PI WT infected kidneys showed scattered foci of inflammation with PMN and macrophages visible in the kidneys (data not shown). At this time, only a few localized inflammatory reactions were observed in mouse kidneys infected with KWN6. After day 3 PI, kidneys infected with both strains had multiple foci of inflammation, with *C. albicans* colonies surrounded by PMN, macrophages, and central necrosis. However, kidneys infected with KWN6 consistently showed less fungal colonization and inflammation than those with WT infection (Fig. 3B, PAS staining). H&E staining confirmed that less pronounced inflammatory responses and tissue necrosis were associated with KWN6 infections (Fig 3B, H&E staining). By day 5 PI, WT infections showed granulomatous kidney lesions with many macrophages and some PMN surrounding the *Candida* colonies, whereas the KWN6 infected kidneys showed better resolution of the initial colonization (data not shown).

For the first day PI, no inflammation was observed in the brain (data not shown), but by day 3 PI the brains of mice infected with WT *C. albicans* showed inflammatory foci containing macrophages, PMN, and *Candida,* whereas brains of mice infected with KWN6 showed fewer inflammatory lesions with less macrophages (data not shown).

### Deletion of *DUR1,2* reduces neutrophil infiltration into infected kidneys

**Figure 4:**
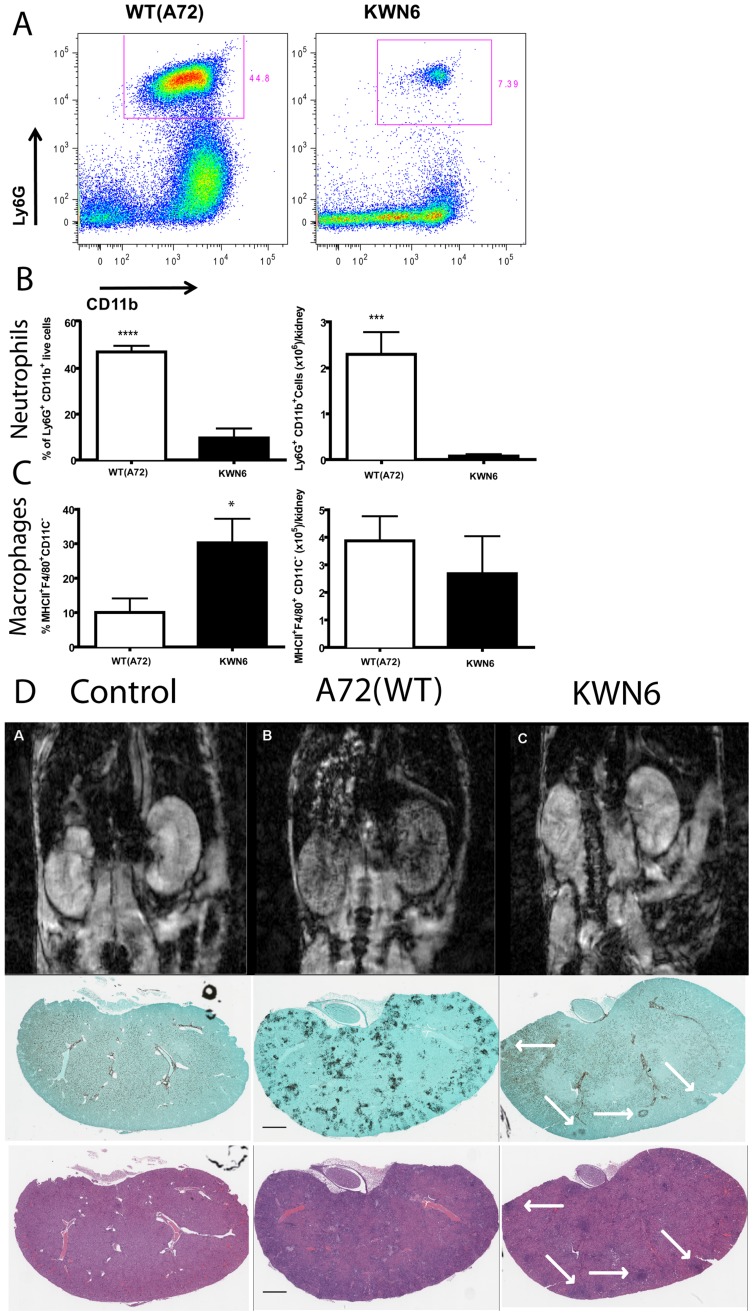
Dur1,2p expression increases inflammatory cell infiltration in infected kidneys. A. Representative FACS plots of kidney neutrophils at day 3 PI with WT and KWN6. **B** Neutrophils expressed as percent of CD45+ cells (left panel) and as absolute numbers per kidney (right). **C**. Macrophages expressed as percent of CD45+ cells (left panel) and as absolute numbers per kidney (right). **D**. Top panels show transverse MR images of 8 week old BALB/c mice 24 h post injection of USPIO agent. Images are representative of at least three non-infected mice per group (left panels), *Candida* infected (middle panels), and infected with KWN6 strain (right panels). Small localized candida colonization by KWN6 is indicated by arrowheads. Massive phagocytic infiltration in kidneys infected with WT is demonstrated by T2* spoiled signal in the kidney cortex and medulla. Lower panels show GMS and H&E stained sections indicating colonization and inflammatory reactions in the respective kidneys imaged in the top panels.

**Figure 5:**
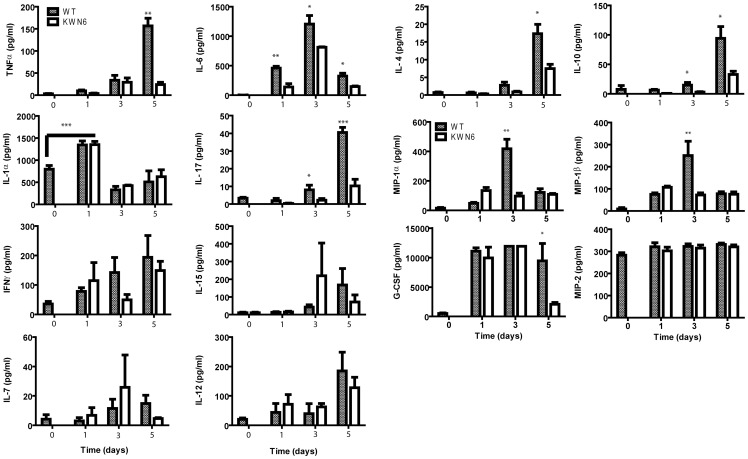
Effects of Dur1,2p expression in *C. albicans* on host serum cytokines and chemokines. Serum levels of the indicated proteins were assessed at 1 to 5 days PI for mice infected iv with WT (gray bars) or KWN6 (open bars). Values at time 0 are mean values determined for sera from 5 control mice, and other data are mean ± standard deviation for 3 mice at each time point. *  =  p<0.05; **  =  p<0.01; ***  = p<0.001.

To quantify the local inflammatory response in infected kidneys, we conducted FACS analysis of infiltrating leukocytes at day 3 (Figs. 4A–C) and 5 PI (data not shown). Examination of neutrophils, monocytes, macrophages, dendritic cells, CD8^+^ T cells, CD4^+^ T cells, and NK cells for their infiltration into kidneys showed that at day 3 PI, mice infected with the urea amidolyase null mutant had significantly lower neutrophil accumulation on both a percentage basis and actual cell counts (6-fold, p<0.0001, and 20-fold, p<0.001, respectively) than did kidneys infected with the WT (Fig 4A and B). In contrast, KWN6 infected kidneys contained a significantly higher percentage of macrophages (p<0.05, Fig. 4C left). This is consistent with the previously observed inability of KWN6 to kill murine macrophages [15]. However, absolute macrophage cell numbers were not significantly different (Fig. 4C right). The levels of kidney colonization by WT *C. albicans* and KWN6 were consistent with the data in Fig. 2A in that kidneys infected with KWN6 had 12-fold lower CFU at 3 days PI (p<0.001, data not shown). These findings also confirmed our histological analysis of fungal invasion by PAS staining and inflammatory response by H&E staining (Fig. 3B). FACS and CFU analyses at day 5 PI did not reveal a significant difference in neutrophil infiltration (data not shown). However, the mutant strain trended towards lower neutrophil accumulation and fungal colonization. FACS analysis at day 5 PI showed that kidneys infected with the mutant strain had a trend toward a higher percentage (26.98% versus 11.32%) and greater absolute numbers (0.591×10^6^ versus 0.099×10^6^ cells/kidney) of neutrophils compared to kidneys infected with the wild-type strain (P = 0.13; data not shown). The day five CFU data may be misleading because histopathology showed considerably less kidney colonization by KWN6 at day 5. This discrepancy may be explained by differences in filamentation, breakage of which during homogenization may lead to an underestimation of CFU in the WT infected kidneys.

### Kidney damage assessed by magnetic resonance imaging (MRI)

As a third method to confirm the differential inflammatory responses to these two strains, we conducted an MRI analysis of kidney function. T2* weighted MRI imaging was performed on infected mice 3 days PI and 24 h after i.v. loading with ultra-small particles of iron oxide (USPIO) as a contrast agent to label phagocytes. Thus, more iron indicates more phagocytes which in turn indicates greater inflammatory reactions in kidney. The top panel of Fig. 4D shows representative MRI sections through the kidneys of infected and non-infected mice, while the lower panels show GMS and H&E stained sections of the same kidneys after imaging. Kidneys of mice infected with the WT strain had widespread phagocyte infiltration of the kidneys as shown by visible perturbation of the MRI T2* signal (Fig. 4D top center panel). GMS and H&E staining of sections from the same kidneys confirmed heavy colonization by *C. albicans* (GMS) and massive inflammatory reactions (H&E) accompanied by numerous PMNs and macrophages. In mice infected with KWN6, the inflammatory response was greatly reduced and more heterogeneous (Fig 4D right panels). GMS and H&E staining of sections from KWN6 infected kidneys confirmed a reduced fungal burden and less inflammation, indicated by white arrows. Thus, by three independent methods, deletion of *DUR1,2* resulted in a reduced and more localized immune response to the infection.

### Deletion of DUR1,2 alters host systemic cytokine and chemokine responses

To further characterize the inflammatory responses in infected mice, we examined serum levels of 10 cytokines and 4 chemokines that may participate in immunity against systemic candidiasis in the mice (Fig. 5). Sera were analyzed pre-infection (day 0) and over the same time frame used for the histopathology and fungal burden analyses. Most dramatically, the proinflammatory cytokine IL-6 was significantly higher in WT infected mice at days 1–5 PI, showing serum increases of 500- to 1200-fold with p<0.01, 0.05 and 0.05, respectively. The importance of IL-6 to the immune response to candidiasis has been noted previously [25], [32]. Serum TNFα levels of WT infected mice were also significantly elevated by day 5 PI (150-fold, p<0.01) as were IL-4, IL 10 and Il-17, which were elevated 18-, 20-, and 10-fold with p<0.05, 0.05 and 0.001, respectively. Similarly, levels of the chemokines MIP-1α and MIP-1β were elevated ca. 100-fold in WT infected mice at day 3 PI with p<0.01. In addition, the pro-inflammatory cytokine IL-1α was elevated significantly in both groups at day 1 PI as was the chemokine G-CSF at all time points examined (Fig. 5). Interestingly, by day 5 PI the KWN6 infected mice had dramatically lower levels of G-CSF (p<0.05) compared with the WT. In contrast, we did not observe significant changes in serum levels for IL-7, IL-12, IL-15, IFNγ, and MIP-2 (Fig. 5).

### Deletion of *DUR1,2* alters inflammatory gene expression in kidneys

Based on the differences in kidney pathology observed during the course of infection with KWN6 versus WT (Figs. 3B and 4D), we examined local host inflammatory responses in infected kidneys using NanoString gene expression profiling for 179 mouse inflammatory genes. Three kidneys of each type were examined at 3 days PI, and 51 genes were identified that showed more than 2.5-fold over- or under-expression with p<0.05. Of these, 19 were over-expressed in WT infected kidneys compared with KWN6 infected kidneys, and 32 were down regulated (Table 1). The fold change ranged from plus 54-fold for *NOS2* to minus 193-fold for *C8a*. We validated the magnitude of these changes using qRT-PCR to quantify changes in mRNA abundance for 9 of the highly up- and down-regulated genes, indicated by asterisks in Table 1 (Figure S1). Consistent with the observed differences in kidney inflammatory responses indicated by H&E and PAS staining (Fig 3), mRNA encoding the proinflammatory marker iNOS (NOS2) showed the highest over-expression in kidneys infected with WT versus the *DUR1,2* deleted strain. This iNOS increase was accompanied by a greater than 14-fold over-expression of mRNAs for the pro-inflammatory mediators IL-1α, IL-1β, and TNF-α. These changes are consistent with the 26-fold increase in c-Fos, which is known to mediate IL-1 induction in an oral epithelial *C. albicans* infection [33]. A 12-fold over-expression of the leukocyte-specific integrin β2 is also consistent with the observed differences in inflammatory cell infiltration. Two members of the MIP chemokine family that play important roles in the recruitment of phagocytes to sites of inflammation [34] were also markedly increased. CCL3 (MIP1α) and CCL4 (MIP1β) were increased 42- and 18-fold, respectively, in WT infected kidneys (Table 1).

In a separate experiment, expression of the same set of genes in KWN6 and A72 infected kidneys were compared with expression in non-infected mouse kidneys by qRT-PCR (Table 2). All of the altered gene expression patterns observed in the first experiment (Table 1) except for CCL3 were confirmed to be significant (p<0.05, Table 2).

**Table 2:** Inflammatory gene expression in qRT-PCR.

Gene	KWN6 infected kidneys	A72 infected kidneys	p value	F C
Nos2	0.8±0.4	12.6±5.5	0.04	16.1
Fos	7.8±3.8	52.8±29.5	0.05	6.8
Ccl3	3.4±1.0	4.7±1.8	0.34	1.4
C8a	−6.3±1.5	−72.9±43.3	0.05	−11.5
MAP2k6	−4.0±1.2	−9.4±1.6	0.01	−2.3
IL-15	−4.0±1.4	−7.8±0.1	0.01	−2.0
Traf2	−0.8±0.1	−1.6±0.3	0.01	−1.9
IL-7	−1.1±0.2	−0.5±0.9	0.01	−0.4

Gene expression in kidneys of mice infected with KWN6 and A72 WT after 3 days is presented as a fold change compared to normal uninfected kidneys. Results are the average of three mice per group with SE.

### Comparison of the inflammatory protein response in infected kidneys

**Figure 6:**
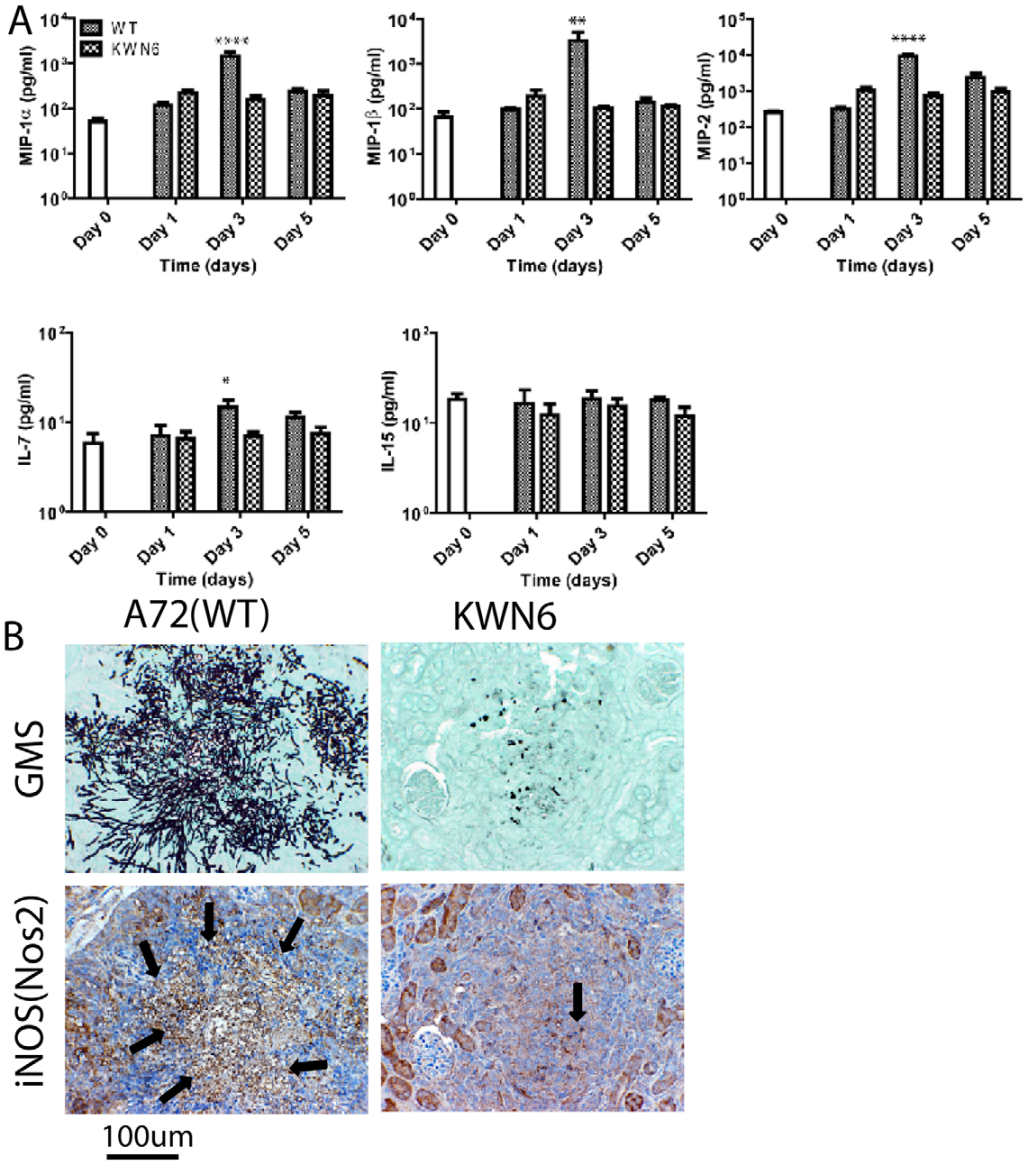
Effects of Dur1,2p expression in local inflammatory markers in infected kidneys. (**A**) Concentrations of cytokines and chemokines in kidney extracts from *C. albicans*-infected mice at 1 to 5 days PI. Values at time 0 are mean values determined for sera from 5 control mice, and other data are mean ± standard deviation for 5 mice at each time point. Mice were infected iv using the wild type strain A72 or the *dur1,2* mutant strain KWN6. Asterisks above an individual bar indicate that the serum value for that protein was significantly greater in mice infected with WT *C. albicans* than in mice infected with KWN6, *  =  p<0.05; **  =  p<0.01; **** = p<0.0001. (**B**) Kidney iNOS expression in cellular infiltrates. Left panels represent WT infected kidneys, and right panels represent KWN6 infected kidneys. Upper panels show *Candida* colonization detected using GMS stain. Lower two panels show micrographs from the same area of an adjacent section stained using an antibody. Arrows indicate regions positive for iNOS (brown stain). Scale bar  = 100 µm.

To determine whether the *DUR1,2*-dependent differences in local inflammatory gene expression result in altered expression of the respective proteins, we analyzed six representative proteins in the kidney extracts (Fig. 6) using the same time points as used for our initial screening. Consistent with the differences in respective mRNA levels, at day 3 PI, we observed significantly up-regulated Ccl3 (MIP1α), Ccl4 (MIP1β), and MIP-2 in kidneys infected with WT relative to those infected with KWN6 (p<0.0001, 0.01, and 0.0001 respectively, Fig. 6A). Comparative IHC using a Nos2 antibody similarly showed that WT infected kidney lesions (Fig. 6B left) had more iNOS specific staining than the KWN6 infected kidneys (Fig. 6B right). In contrast, the decreases in IL-7 and IL-15 mRNA levels did not correlate with changes in IL-15 and IL-7 protein expression (Fig. 6A).

### Global inflammatory pathways regulated by Dur1,2p

To better understand potential relationships between the observed changes in inflammatory genes, the kidney mRNA expression data was analyzed using MetaCore software. Five host signaling pathways were significantly altered when *DUR1,2* was deleted (Figure S2). The two pathways achieving highest significance were the classical and lectin-induced complement pathways (Figure S2). The complement system is a major effector in protective humoral immunity against microorganisms. Activated C3 and C5 convertases interact sequentially to form a membrane attack complex that creates pores in the cell membrane and induces cell lysis [35]. Infected WT kidneys had down regulated C2 (4-fold) and C8a (193 fold). The most down-regulated gene, C8a, encodes two subunits of the final membrane attack complex. The decrease in C8a was surprising given that IL-1β, which was elevated in the same kidneys, induces C8a in other organs such as liver [36]. These urea-dependent changes in complement function in the kidneys may account for the known ability of WT *C. albicans* to limit host humoral immunity [37].

The IL-15 pathway and several downstream genes involved in this pathway including TRAF2, a signal transducer that associates with the IL-15 receptor [38], were significantly down regulated in mouse kidneys infected with WT strain (Table 1,2 and Figure S1). IL-15 is a pleiotropic cytokine involved in proliferation, differentiation, immune responses, and cell survival [39]. Dysregulation of IL-15 is emerging as important for the pathogenesis of auto-immune diseases and host immune responses to cancer [40], and thus IL-15 may exert a protective role against candidiasis by regulating T-cell differentiation, phagocytosis, neutrophil stimulation, or monocyte migration.

The IL-1 signaling pathway also achieved significance. IL-1 stimulates a broad spectrum of immune and inflammatory responses [41]. Kidneys of mice infected with the WT strain up-regulated IL-1α (15 fold), IL-1β (14 fold), and the interleukin 1 receptor antagonist IL1RN (37-fold) relative to those infected with the strain lacking *DUR1,2*. These changes are consistent with the increased expression of the IL-1-regulated transcription factor MAFF and inflammation in the WT infection [42]. Additional elements of the IL-1 pathways including TNFα, TGFβ and iNOS were also significantly upregulated. TNFα and IL-1β are inducers of *Nos2* gene expression [43], [44], which is consistent with the observed changes in iNOS and protein and mRNA expression. Although the high levels of NO produced by iNOS have potent anti-microbial activity, *Candida* may be resistant to this host defense due to expression of *YHB1,* which converts NO to nontoxic nitrite [45].

The direct interaction algorithm identified interactions among 8 of the up-regulated genes that mediate pro-inflammatory responses (Figure S3a). Similarly, 17 of the down regulated genes have known direct interactions (Figure S3b).

## Discussion

Our results show that deletion of *DUR1,2* significantly reduces virulence in a mouse model of disseminated candidiasis and that virulence is restored when *DUR1,2* is reconstituted. The decreased pathogenicity of KWN6 (*dur1,2*Δ*/dur1,2*Δ) was evidenced by decreased colonization of the brain and kidneys, decreased kidney damage, and a decrease in the extent and type of the inflammatory response mounted by the infected mice. In particular, cytokine and chemokine concentrations in serum and kidneys were dramatically altered. Taken together, these results indicate that *DUR1,2* and the encoded urea amidolyase act as a virulence factor for murine disseminated candidiasis and potentially for other infections caused by *C. albicans*. From a more global perspective, they suggest an important role of urea availability in determining the location and severity of *C. albicans* infections. Notably, the organs in which *C. albicans* can establish persistent colonization are those with the highest urea contents [27], [28], [29]. Urea in the kidney is concentrated along the renal corticomedullary axis by the function of several active urea transporters. In healthy kidneys urea is further concentrated and sequestered in collecting ducts, but fungal infection, tissue necrosis, and the host inflammatory response may cause accumulation of urea [29], which further enhances virulence.

In addition to providing nitrogen for growth, our data indicate that urea metabolism by *C. albicans* has profound effects on the host inflammatory and immune responses. In addition to its established role in escape from macrophages, urea metabolism has both local and systemic effects on the host immune response. The long blood circulating time and progressive phagocytic uptake of USPIO particles [46] enabled us to use MRI imaging as a tool to non-invasively follow phagocyte recruitment and tissue inflammation in infected mice. These imaging results were validated by histopathology, immunohistochemistry and expression of inflammatory cytokines and the activated phagocytic marker iNOS. The 50-fold increase in iNOS mRNA expression in WT *C. albicans* infected kidneys could result from increased neutrophil or M1 macrophage infiltration [47].

Increased survival of mice infected with the *dur1,2* mutant may be explained in part by its inability to persist beyond 7 days PI in kidneys of infected mice. A number of previous pathogenesis studies indicated that kidney is the key battleground for survival of candidiasis [28], [48], [49], [50]. Approximately 90% of *C. albicans* cells are cleared from mouse blood within 3 min of tail vein injection [51]. In addition to kidney, other organs including brain, liver, lung, and spleen are initially colonized by *C. albicans* in immunocompetent mice, but except for kidney and to a lesser degree brain, all the organs are cleared by 4 days PI [20], [49]. Colonization selectively persists in the kidney in both mice and humans [52].

*C. albicans* infection is associated with increased levels of pro inflammatory monocyte derived cytokines such as TNFα, IL-1, and IL-6 [53] as well as high IL-10, which contribute to the suppression of immunity against candidiasis [54]. In addition, Th2 responses, indicated by high levels of IL-4, are detrimental to a host/patient with disseminated candidiasis [55], [56]. We recently reported that the decreased virulence of a *C. albicans hmx1* mutant is associated with alterations in systemic cytokine levels that indicate a more balanced host immune response [6]. We propose that the loss of urea degradation that results from deletion of *DUR1,2* has a similar balancing effect on systemic host immunity.

Urea metabolism by *C. albicans* in the kidney also exacerbates local host inflammatory gene responses. Several of these chemokines attract neutrophils, and their persistence causes collateral damage to host tissue that may lead to tissue necrosis and impaired kidney function. The improved kidney function and reduced *Candida* colonization and inflammatory reactions associated with KWN6 infection suggest that the role of urea metabolism in colonization and inflammation in the kidney involves the control of local inflammatory reactions, particularly neutrophils at early time points. For, example, MIP2 and IL-7 were up-regulated locally in WT infected kidneys compared with the KWN6 infected kidneys but were not altered systemically. This more balanced immune response may prevent the chronic stage of colonization of the renal medulla and pelvis [6], [31], contributing to the higher survival in mice infected with the *DUR1,2* mutant relative to the WT strain.

CCL3 (MIP1α) and CCL4 (MIP1β) are chemokines that promote recruitment of neutrophils, macrophages and other leukocytes to sites of inflammation [34]. Mice lacking CCL3 have a reduced inflammatory response to influenza virus and are resistant to coxsackievirus-induced myocarditis [57]. Although these chemokines can play positive roles in resolving acute inflammation, they are increasingly recognized as detrimental for chronic inflammation, and therapeutic inhibitors of MIP1inflammatory chemokines are being developed to treat diseases associated with chronic inflammation. The increased expression of these chemokines in kidneys infected with WT *C. albicans*, therefore, could play a role in the strong inflammatory responses we observed. In the absence of Dur1,2p activity, inflammation and expression of CCL3 and, more reproducibly, CCL4 was markedly decreased. Altered expression of MIP1 chemokines was recently associated with virulence in a different *C. albicans* mutant [6]. Both chemokines showed decreased circulating levels in mice infected with *C. albicans* lacking *HMX1*, and their expression was regulated by the immunosuppressive carbon monoxide (CO) produced by Hmx1p. Decreased kidney expression of CCL3 and CCL4 was also noted in mice infected with a *pmr1*Δ mutant of *C. albicans* with decreased virulence [58], [59]. Determining how Dur1,2p expression increases CCL3 and CCL4 expression in infected kidneys, therefore, is an important topic for future research.

Given the importance of the kidney for concentrating and excreting urea, we propose that urea metabolism to ammonia and CO_2_ by Dur1,2p plays a role in the persistence of *C. albicans* in this organ. CO_2_ is known to inhibit macrophage clearance of *C. albicans* [15], and so may limit the efficacy of the macrophages recruited into infected kidneys. NH_3_ has been considered as a virulence factor for *C. albicans* [60], and its importance for virulence is well established for *Helicobacter pylori* [61]. NH_3_ produced by this bacterial pathogen via urease plays a critical role in controlling local pH and facilitating invasion through the gastric mucosa. A second enzymatic pathway that produces NH3 by deamidation of asparagine or glutamine is also essential for colonization of the stomach environment by *H. pylori* [62]. NH3 plays an additional role in virulence of *H. pylori* by enhancing host cell apoptosis through its modulation of endocytic vesicle trafficking [63]. Furthermore, NH_3_ is increasingly recognized as an important signaling molecule in cellular responses to stress [64], [65]. In the context of inflammation, elevated NH_3_ levels inhibit neutrophil chemotaxis [66], phagocytosis, and degranulation while also stimulating spontaneous oxidative bursts [67]. NH_3_ also inhibited the capacity of neutrophils to engulf bacteria [68]. Notably, the latter study implicated the p38 pathway in this neutrophil dysfunction, and we observed differential expression 4 MAP kinases that are direct (Map2k6 and Map2k4) or indirect activators of p38 (Map3k5 and Map3k7). Therefore, NH_3_ production by Dur1,2p may mediate some of the changes in host gene expression and neutrophil recruitment between mice infected with WT and the *dur1,2* mutant.

Our results are also compatible with the specific requirement of *DUR1,2* for germ tube formation in the macrophage [15] and evidence that that urea metabolism via Dur1,2p provides ammonia for external alkalinization of the *C. albicans* environment [60], thus permitting germ tube formation and escape from the phagolysosome. Despite its defect in hyphal-dependent escape from macrophages [15], the *dur1,2* mutant can make hyphae except when driven exclusively by arginine in macrophages or in response to urea in vitro. This selective defect in hyphal differentiation may contribute to the lower abundance of filaments in kidneys infected with the mutant strain at 3 and 5 days PI.

Taken together, our present and previous results strongly indicate that expression of Dur1,2p enhances kidney neutrophil infiltration but limits phagocytic clearance of *C. albicans*. The persistence of neutrophils late in the course of infection correlates with more tissue damage and immunopathology that leads to higher mortality [69]. Mice infected with a *DUR1,2* deleted strain show greater survival and a more balanced immune response with less persistent neutrophil infiltration into the kidney. Thus, Dur1,2p appears to act locally in the kidney to create a pro-inflammatory state that is detrimental to the host. This could account for the greater renal malfunction and mortality in mice infected with WT *C. albicans* compared with the mutant lacking Dur1,2p. Correspondingly, a pharmacological inhibitor of Dur1,2p could improve patient survival of disseminated candidiasis by improving the innate immune response to kidney infections.

## Supporting Information

Figure S1 **Effect of** ***DUR1,2*** **on inflammatory gene expression of mouse kidneys.** mRNA abundance was determined by qPCR using RNAs prepared from WT *C. albicans* infected kidney and KWN6 infected kidney. Fold change in mRNA expression normalized to HPRT mRNA abundance is shown for WT infected kidneys compared with KWN6 infected kidneys. Experiments were performed in triplicate; error bars, SEM. Positive numbers indicate higher gene expression in WT infected kidneys, and negative numbers represent higher expression in KWN6 infected kidneys.
(TIF)

Figure S2 **Summary of Genego pathway maps.** The twenty most statistically significant pathway maps generated by MetaCore algorithms are shown.
(TIF)

Figure S3 **Direct functional interactions between genes shown in** **Fig.** **3** **.** **a**. genes up regulated in kidney infected with WT compared with KWN6 infected kidneys. **b**. Genes down regulated in kidneys infected with WT compared with KWN6 infected kidneys. Key: receptor ligands (green symbols), transcription factors (red symbols), enzymes (yellow symbols), receptors (blue symbols), and protein kinases (orange symbols). Green arrows indicate positive effects, red arrows indicate negative effects, and grey arrows indicate unspecified link or technical link. A yellow dot on the middle of the symbol indicates related proteins or compounds that are connected into groups.
(TIF)

Table S1 Sequences of Synthetic oligonuclotides used in this study.
(DOCX)
