# Supplementary figures and images for: Urea Amidolyase (DUR1,2) Contributes to Virulence and Kidney Pathogenesis of Candida albicans
Source: PLoS One. 2012 Oct 29;7(10):e48475. doi: 10.1371/journal.pone.0048475 (PMC3483220; doi:10.1371/journal.pone.0048475)

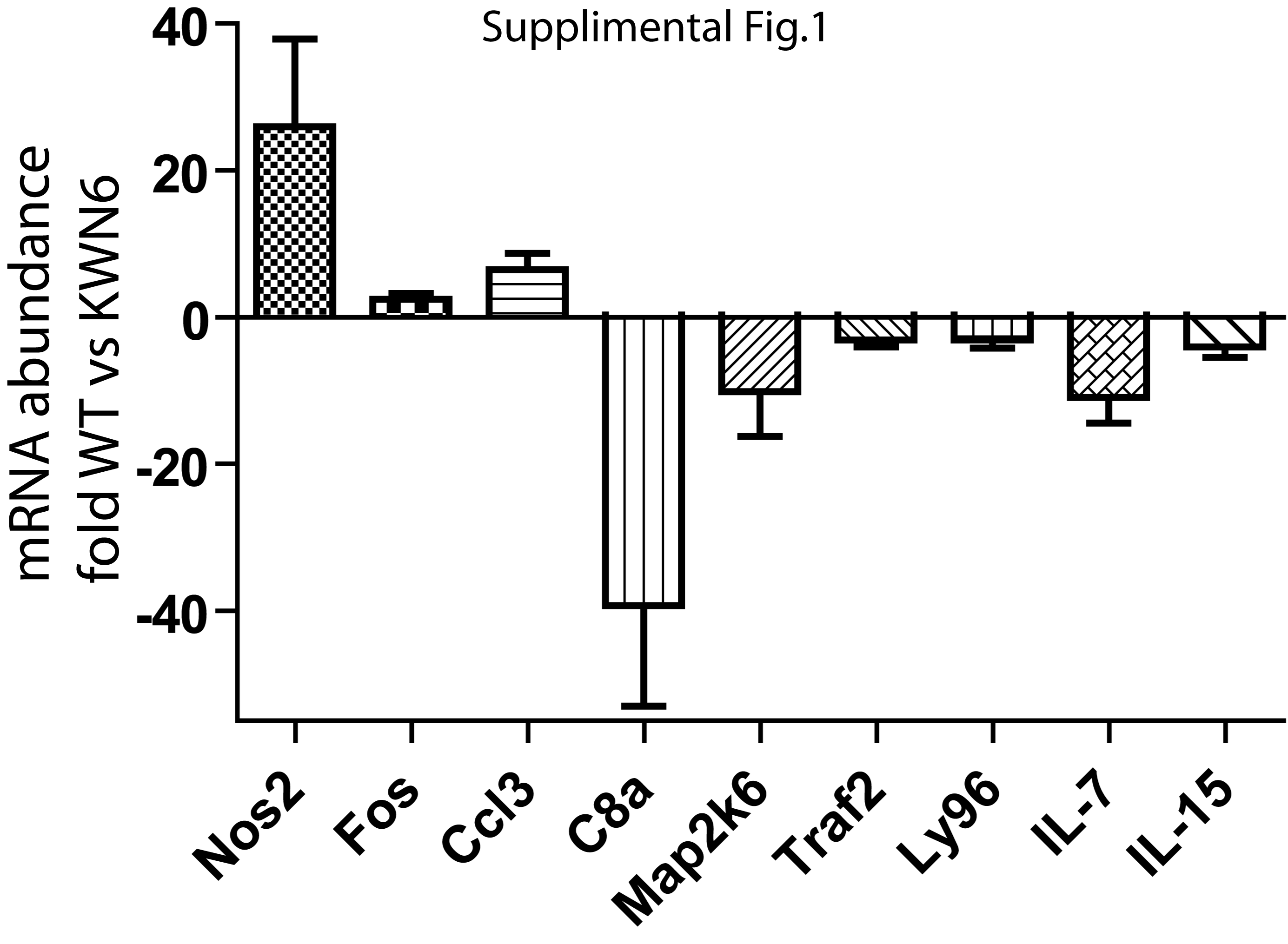

Supplement: Figure S1 — Effect of DUR1,2 on inflammatory gene expression of mouse kidneys. mRNA abundance was determined by qPCR using RNAs prepared from WT C. albicans infected kidney and KWN6 infected kidney. Fold change in mRNA expression normalized to HPRT mRNA abundance is shown for WT infected kidneys compared with KWN6 infected kidneys. Experiments were performed in triplicate; error bars, SEM. Positive numbers indicate higher gene expression in WT infected kidneys, and negative numbers represent higher expression in KWN6 infected kidneys. (TIF) [file pone.0048475.s001.tif]

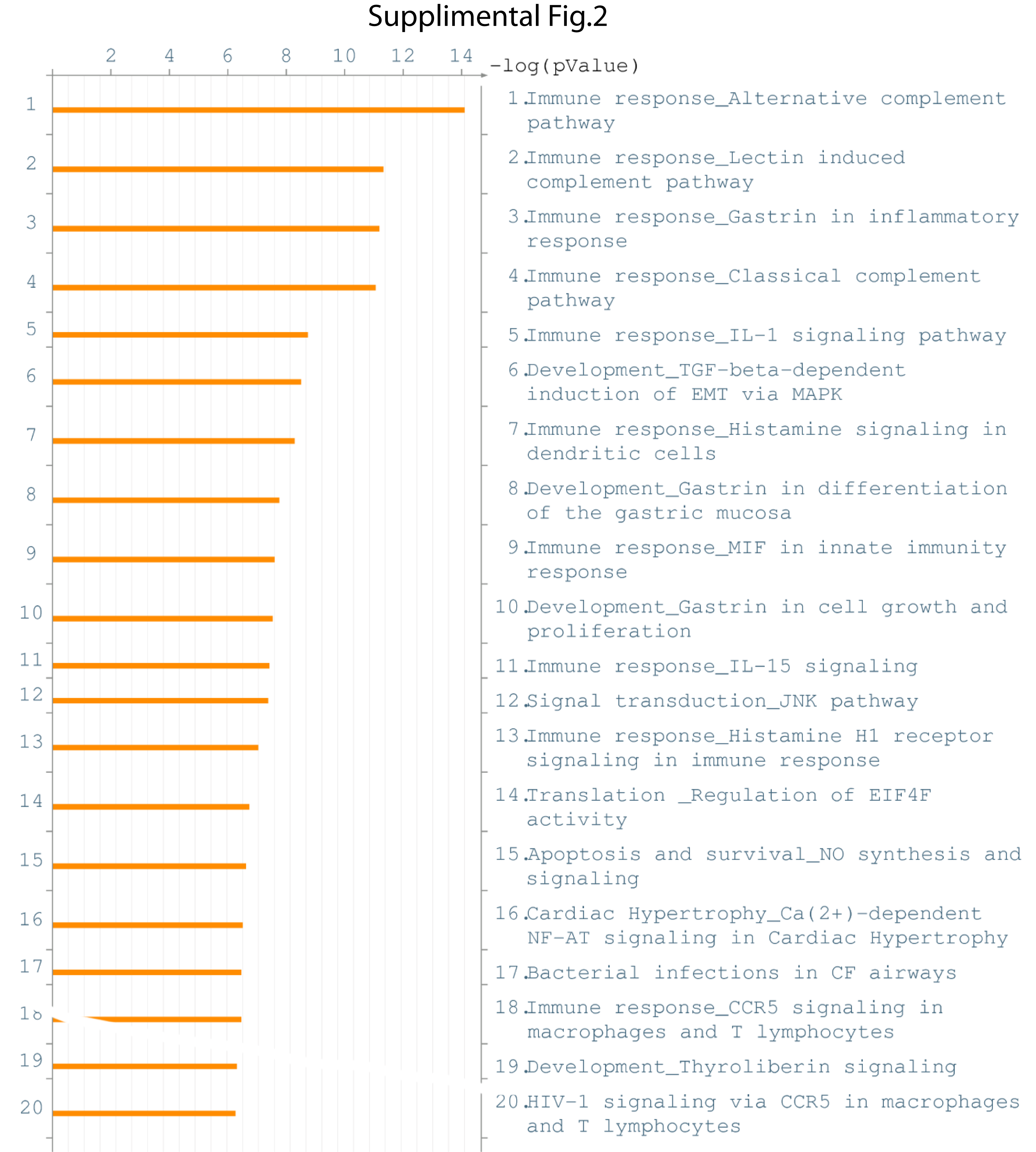

Supplement: Figure S2 — Summary of Genego pathway maps. The twenty most statistically significant pathway maps generated by MetaCore algorithms are shown. (TIF) [file pone.0048475.s002.tif]

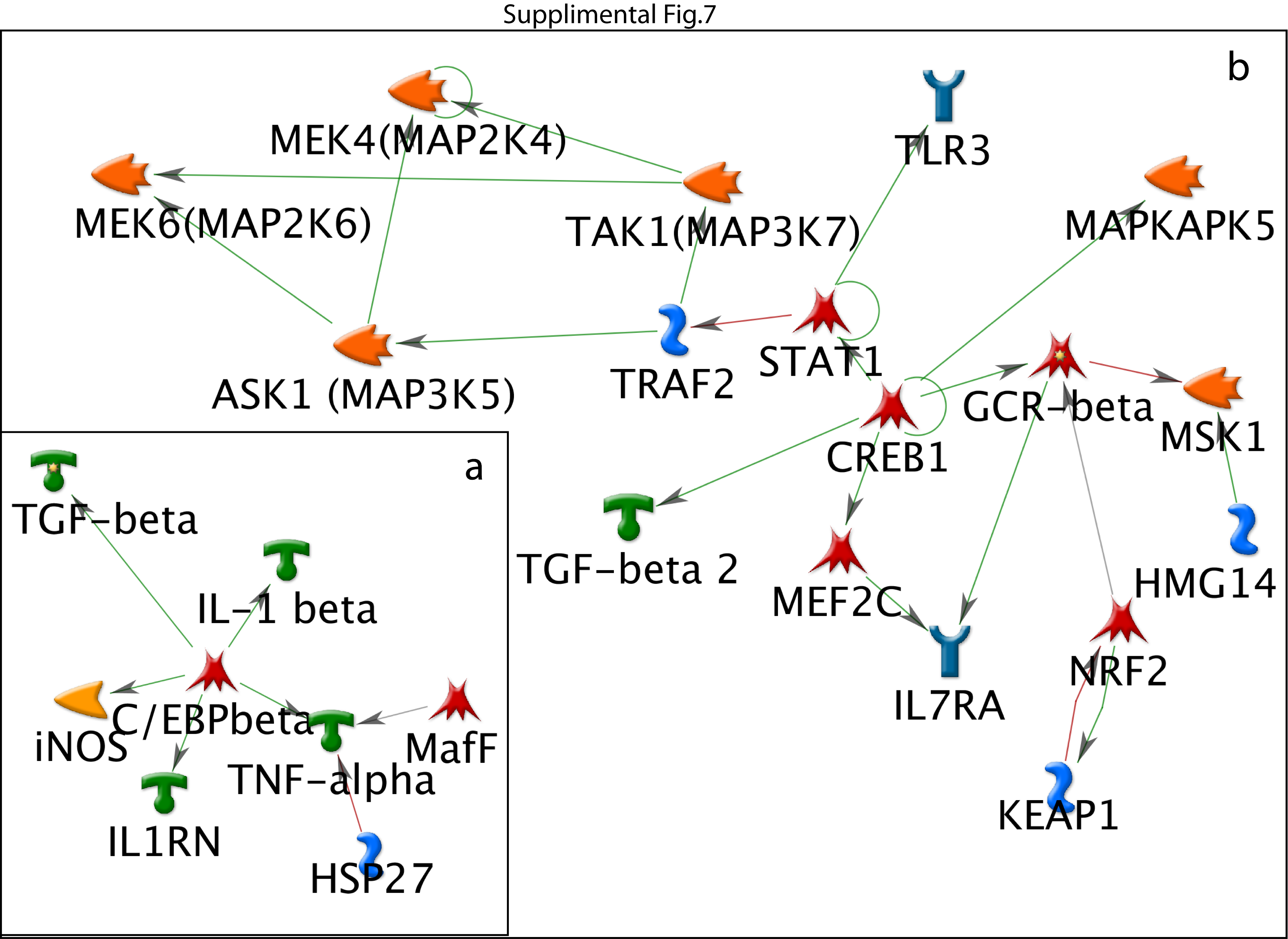

Supplement: Figure S3 — Direct functional interactions between genes shown in Fig. 3 . a. genes up regulated in kidney infected with WT compared with KWN6 infected kidneys. b. Genes down regulated in kidneys infected with WT compared with KWN6 infected kidneys. Key: receptor ligands (green symbols), transcription factors (red symbols), enzymes (yellow symbols), receptors (blue symbols), and protein kinases (orange symbols). Green arrows indicate positive effects, red arrows indicate negative effects, and grey arrows indicate unspecified link or technical link. A yellow dot on the middle of the symbol indicates related proteins or compounds that are connected into groups. (TIF) [file pone.0048475.s003.tif]
