# Supplementary material for: Urea Amidolyase (DUR1,2) Contributes to Virulence and Kidney Pathogenesis of Candida albicans
Source: PLoS One. 2012 Oct 29;7(10):e48475. doi: 10.1371/journal.pone.0048475 (PMC3483220; doi:10.1371/journal.pone.0048475)
Supplement: Table S1 — Sequences of Synthetic oligonuclotides used in this study. (DOCX) [file pone.0048475.s004.docx]

Supplemental Table.1

Sequences of Synthetic oligonuclotides used in this study

| Sequence | Name | Reference |
| --- | --- | --- |
| 5’- CCATGAAGTCTGGGAAGAGG -3’ | mNos2RT-Lt | This study |
| 5’- TCGCTAGCCCTGTGTCTACC -3’ | mNos2 RT-Rt | This study |
| 5’- GACCAGGCCTTGACTCACAT-3’ | mFos RT-Lt | This study |
| 5’- CCAGTCCTCACCTCTTCCAG-3; | mFos RT-Rt | This study |
| 5’- GATGAATTGGCGTGGAATCT-3; | mCcl3 RT-Lt | This study |
| 5’- ATGAAGGTCTCCACCACTGC-3’ | mCcl3 RT-Rt | This study |
| 5’- TTCGTCATTTTTCCCAGTCC-3’ | mC8a RT-Lt | This study |
| 5’- GGTGCCTATCTGAACCTGGA-3’ | mC8a RT-Rt | This study |
| 5’- CCCCTTTGGAGAGGAAAAAG-3’ | mMap2k6 RT-Lt | This study |
| 5’- ACTGGTCGACCCTACTGTGG-3’ | mMap2k6 RT-Rt | This study |
| 5’- GAAGGCCAGTCACTTTGCTC-3’ | mTraf2 RT-Lt | This study |
| 5’- AGCTCCTGCTGTCTCCTCTG-3’ | mTraf2 RT-Rt | This study |
| 5’- GGCACAGAACTTCCTTACGC-3’ | mLy96 RT-Lt | This study |
| 5’- CCAATGGATTTGTGCATGTT-3’ | mLy96 RT-Rt | This study |
| 5’- TGGTTCATTATTCGGGCAAT-3’ | mL-7 RT-Lt | This study |
| 5’- ATCCTTGTTCTGCTGCCTGT-3’ | mIL-7 RT-Rt | This study |
| 5’- TTCGTCCAACTCTGCAACTG-3 | mIL-15 RT-Lt | This study |
| 5’- CAGGTCCTCCTGCAAGTCTC-3’ | mIL-15 RT-Rt | This study |
